# P-223. Risk Factors for Community-Associated Clostridium difficile Infection: A Retrospective Study

**DOI:** 10.1093/ofid/ofae631.427

**Published:** 2025-01-29

**Authors:** Barak Mizrahi, Miriam Weinberger, Shira Lidar, Shirley Shapiro Ben David

**Affiliations:** KI Research Institute, Tel Aviv, Tel Aviv, Israel; Tel Aviv University, Tel Aviv, Tel Aviv, Israel; Maccabi Healthcare Services, Tel Aviv, Tel Aviv, Israel; Maccabi Healthcare Services, Tel Aviv, Tel Aviv, Israel

## Abstract

**Background:**

Community-associated Clostridium difficile infection (CA-CDI) has emerged as a significant public health concern in recent years. The objective of this study was to evaluate the risk factors for CA-CDI including antibiotics-specific regimen and duration.

**Table 1:**
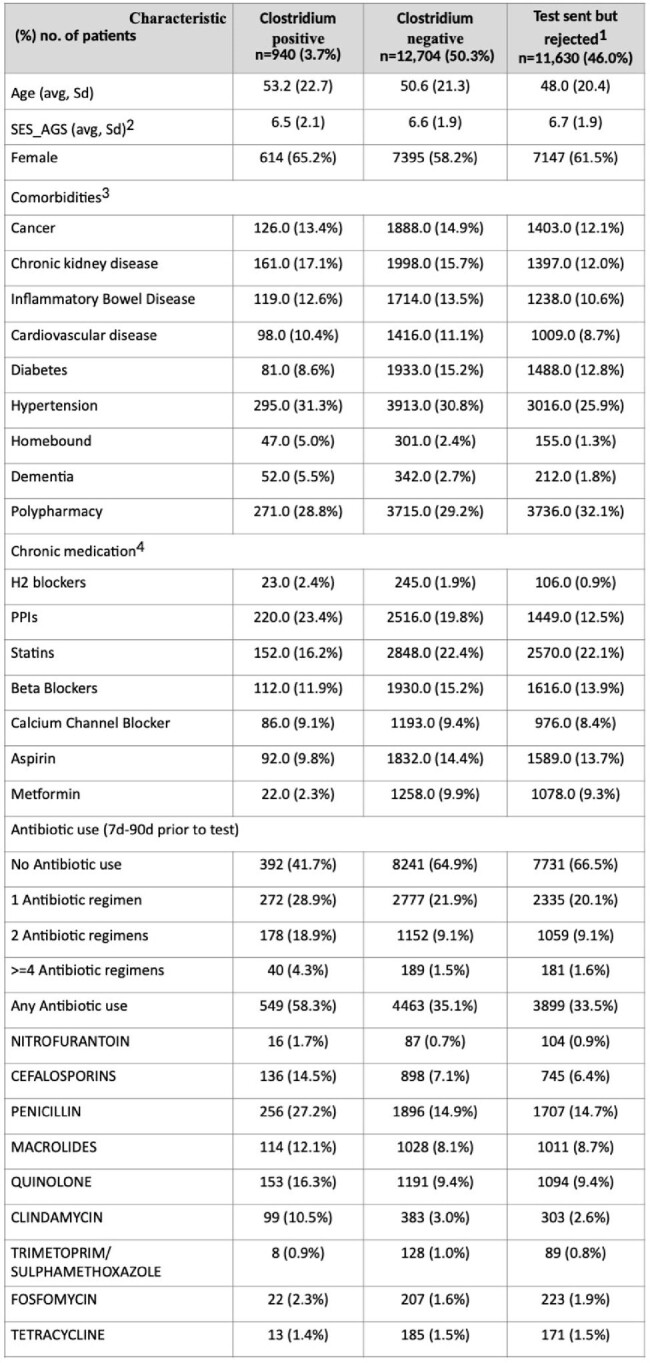
Baseline characteristics of study cohort 1- Tests were sent but rejected by the laboratory due to non-diarrheic stool; 2- SES socioeconomic status; 3- Based on registries; 4- minimum three sequential monthly purchases

**Methods:**

Data from a large health maintenance organization was extracted between 2001-2023. The study population included patients who were sent to their first Clostridium difficile (CD) test ever, with no records of hospital admission within 84 days preceding the test. Patients with positive CD test were considered CA-CDI cases. They were compared to patients with negative tests and to patients whose test was rejected due to non-diarrheic stool. Correlates of CD were detected using multivariate logistic regression. Inverse probability weighting was used to examine the associations between exposure to different antibiotic regimes, types or durations, after controlling for potential confounders.

**Figure 1:**
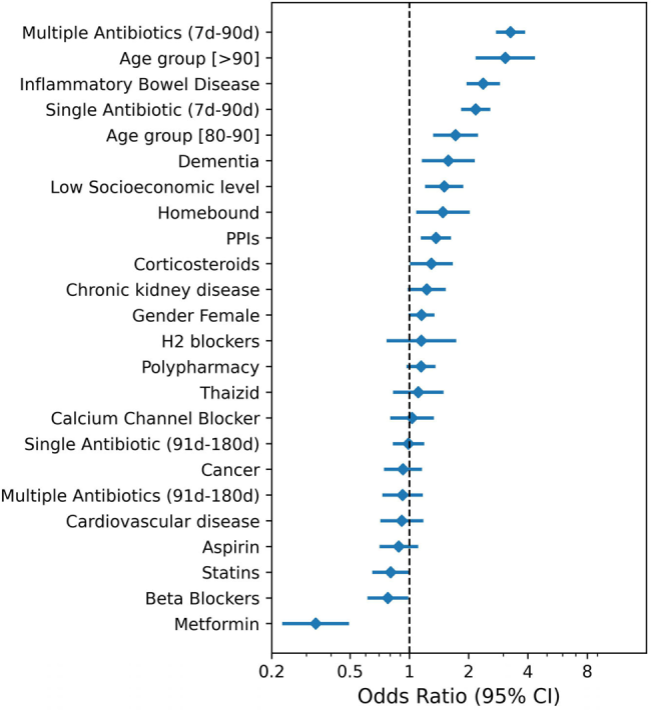
Risk factors for CA-CDI CA-CDI, Community-associated Clostridium difficile infection

**Results:**

During 2001-2023 25,274 patients with first CD tests were identified, of whom 940 (3.7%) were CA-CDI cases, 12,704 (50.3%) were CD test-negative, and 11,630 (46.0%) patients had rejected tests (Table 1). The mean age was 53.2 (22.7), 50.6 (21.3), and 48.0 (20.4), respectively, and the number of females was 614 (65.2%), 7395 (58.2%), and 7147 (61.5%), respectively. Various conditions or exposures were associated with increased risk for CA-CDI, including inflammatory bowel disease, dementia, homebound status, and proton pump inhibitor) (use, whereas metformin was associated with reduced risk of CA-CDI (Figure 1). The number of different antibiotic agents was a significant risk factor, with risk increasing twofold for 1 agent vs. none, and additional increments with each additional antibiotic (Figure 2). When the exposure was five days the risk was elevated only for clindamycin and quinolones, while with ≥10 days-exposure, the risk was significantly elevated for all antibiotics agents (Figure 3).

**Figure 2:**
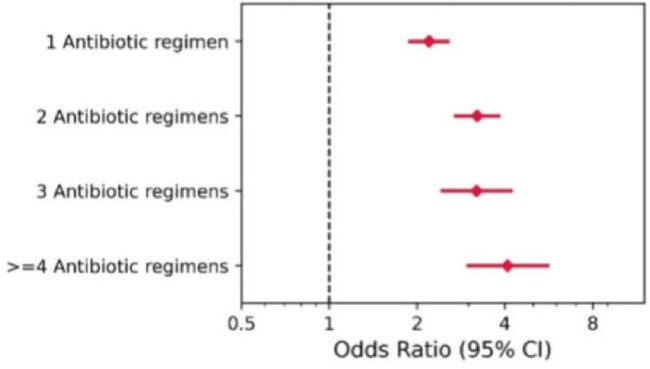
Risk of CA-CDI according to number of antibiotic exposure (7-90 days before infection)

**Conclusion:**

This study provides insights into the risk factors for CA-CDI in the community setting and underscores the importance of judicious antibiotic prescribing practices and specifically longer duration of antibiotic use.

**Figure 3:**
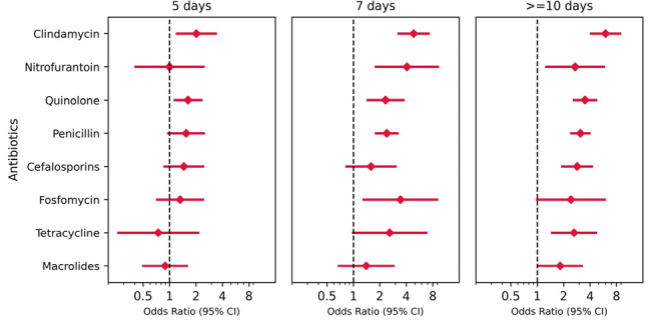
Risk of CA-CDI according to antibiotic exposure*- regimen and duration *7-90 days before infection

**Disclosures:**

**Shirley Shapiro Ben David, MD**, GSK: Lecturer|Pfizer: Grant/Research Support|Pfizer: Lecturer

